# Separase and Roads to Disengage Sister Chromatids during Anaphase

**DOI:** 10.3390/ijms24054604

**Published:** 2023-02-27

**Authors:** Marketa Konecna, Soodabeh Abbasi Sani, Martin Anger

**Affiliations:** 1 Department of Genetics and Reproduction, Veterinary Research Institute, 621 00 Brno, Czech Republic; 2 Institute of Animal Physiology and Genetics, Czech Academy of Science, 277 21 Libechov, Czech Republic; 3 Faculty of Science, Masaryk University, 602 00 Brno, Czech Republic

**Keywords:** separase, chromosome division, segregation errors, cohesin, securin, Cyclin B1, CDK1, Sgo2, Mad2, aneuploidy

## Abstract

Receiving complete and undamaged genetic information is vital for the survival of daughter cells after chromosome segregation. The most critical steps in this process are accurate DNA replication during S phase and a faithful chromosome segregation during anaphase. Any errors in DNA replication or chromosome segregation have dire consequences, since cells arising after division might have either changed or incomplete genetic information. Accurate chromosome segregation during anaphase requires a protein complex called cohesin, which holds together sister chromatids. This complex unifies sister chromatids from their synthesis during S phase, until separation in anaphase. Upon entry into mitosis, the spindle apparatus is assembled, which eventually engages kinetochores of all chromosomes. Additionally, when kinetochores of sister chromatids assume amphitelic attachment to the spindle microtubules, cells are finally ready for the separation of sister chromatids. This is achieved by the enzymatic cleavage of cohesin subunits Scc1 or Rec8 by an enzyme called Separase. After cohesin cleavage, sister chromatids remain attached to the spindle apparatus and their poleward movement on the spindle is initiated. The removal of cohesion between sister chromatids is an irreversible step and therefore it must be synchronized with assembly of the spindle apparatus, since precocious separation of sister chromatids might lead into aneuploidy and tumorigenesis. In this review, we focus on recent discoveries concerning the regulation of Separase activity during the cell cycle.

## 1. Introduction

Since DNA replication occurs during S phase, sister chromatids are connected along their longitudinal axes, and this connection allows them to be properly segregated at anaphase. The ties between sister chromatids are formed by a protein complex called cohesin [1,2,3] (reviewed in [4,5,6]). This complex is not only crucial for sister chromatid cohesion, but it is also essential for homologous recombination and DNA repair, and the regulation of gene expression and structuring of chromatin (reviewed in [7,8,9,10]). The cohesin complex core proteins form a ring-shaped complex, which consists of heterodimers of Structural maintenance of chromosomes proteins 1 and 3 (Smc1, Smc3), Sister chromatid cohesion protein 1 (Scc1) or Meiotic recombination protein Rec8 homolog (Rec8), kleisin subunits and Stag1-3 (SA1, SA2 or SA3) subunits. Since the cohesion fulfills diverse functions, there are other proteins interacting with the cohesin complex, which are required for cohesion loading or displacement, such as, for example, Precocious dissociation of sisters 5 (Pds5) [11], Sister chromatid cohesion protein 3 (Scc3) [12], Wings apart-like protein homolog (Wapl, protein was originally identified in Drosophila) [13] and many others [5,14,15]. Cohesin complex is loaded onto chromosomes during late telophase and subsequent G1 by activity of the Sister chromatid cohesion protein 2/4 (SCC2/4) complex (in human Nipped-B-like protein/MAU2 sister chromatid cohesion factor, NIPBL-MAU2) [16,17,18,19]. Acetylation of Smc3 during the S phase then facilitates recruitment of another cohesin regulator called Sororin [20], which opposes Wapl in its cohesin-releasing activity [21]. Sororin is therefore important for cohesion maintenance until mitotic entry. Additionally to the cohesion, the sister chromatids are held together by topological intertwines of DNA, also known as catenanes [22,23]. However, it was shown that the stability of concatenations between sister chromatids also requires cohesion [24]. After entry into mitosis, the DNA catenations are resolved by Topoisomerase II and condensin complex before anaphase [25,26] (reviewed in [27]). Currently published surprising results showed that some connections between sister chromatids are preserved even after anaphase and they are important for chromosome movements after the separation of chromatids [28].

**Figure 1:**
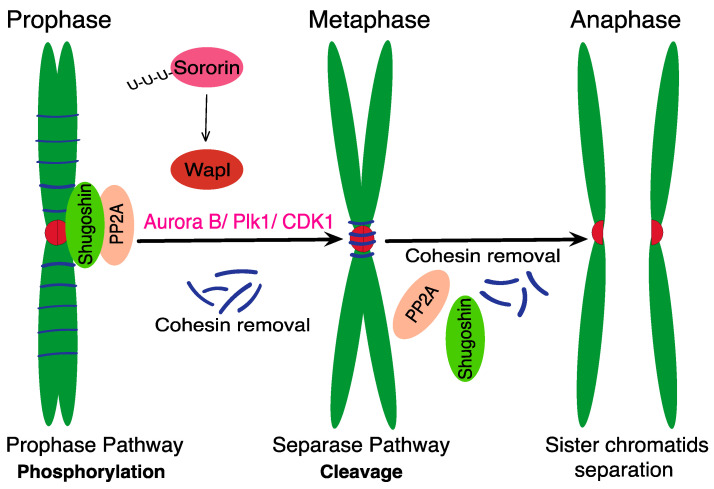
Two-step cohesin removal in mitosis. The prophase pathway removes the cohesin complex from chromosome arms [29]. Inactivation and destruction of Sororin by APC/C Cdh1 activates Wapl protein and leads to loss of cohesion at chromosome arms [21,31]. The PP2A and Shugoshin protect the centromeric cohesin [32,33,34] by counteracting phosphorylation caused by Plk1, CDK1 and Aurora B [35,36,37]). The separation of sister chromatids during anaphase requires the cleavage of remaining cohesion by Separase.

To allow chromosome segregation in anaphase, the cohesin, holding together sister chromatids, must be removed from chromosomes. In vertebrate cells, this process is divided into two waves (Figure 1). After entry into mitosis, during prophase, the bulk of cohesion is removed from chromosome arms by a mechanism called the prophase pathway [29]. Liberation of chromosome arms causes the typical X shape of metacentric chromosomes, visible on chromosome spreads from metaphase cells. The removal of cohesion in prophase does not involve its cleavage. It requires, however, inactivation of Sororin by Anaphase-promoting complex/cyclosome Cdc20 homolog 1 (APC/C Cdh1)-dependent ubiquitination and proteolytic destruction, which in turns activates the Wapl protein. Other factors, such as the activity of kinases Plk1, CDK1 and Aurora B, are also required (reviewed in [5,30]). The prophase pathway in vertebrates is an essential process, and deletion of the gene encoding Wapl was shown to be embryonically lethal [31]. The prophase pathway removes the cohesin complex from chromosome arms almost entirely, leaving only small traces at the centromeres. However, this amount is sufficient to keep sister chromatids together until anaphase. The protection of cohesion located there is facilitated by proteins called Shugoshins (Sgo1/2) [32,33,34]. Together with Protein phosphatase 2A (PP2A), they colocalize to the centromere, where PP2A protects the centromeric cohesion and Sororin by opposing phosphorylation induced by Plk1, CDK1 and Aurora B kinases [35,36] (reviewed in [37]).

The second wave of cohesin removal is scheduled to anaphase. During anaphase, sister chromatids are separated and then segregated to the opposite poles of the spindle. The separation and poleward movement of sister chromatids is triggered by the removal of cohesion, holding together sister chromatids. In yeasts, because of the absence of prophase pathways, the cohesin is preserved and at this point still connects sister chromatids along their entire longitudinal axes, whereas in vertebrate cells, the remaining cohesion after the prophase pathway is located mostly in the vicinity of the centromeres. Similarly to the yeast, in vertebrate female germ cells, the prophase pathway against cohesion holding together sister chromatids (and therefore also bivalents after chiasmata formation) is almost entirely suppressed [38]. Wapl-dependent cohesin removal in mouse meiosis I is however also operational, but its activity is aimed at dismantling only cohesion-containing Scc1 [39]. The pathway removing cohesion at anaphase is conserved from yeasts to vertebrates, and it requires cohesion cleavage, which finally separates sister chromatids [29,40,41,42,43]. The protease carrying this function was first discovered in budding and fission yeast, as Extra spindle poles-like protein 1 (Esp1), respective cut1 mutants, which exhibited problems during chromosome segregation and later were called Separase [44,45]. Separase cleaves the cohesin kleisin subunits Scc1 during mitosis [29,41] or Rec8 during meiosis [38,46]. The cleavage of Scc1 or Rec8 by Separase is dependent on phosphorylation by Cell division cycle 5/Polo-like kinase 1 (CDK5/Plk1) in mitosis [47,48], or by Casein Kinase 1 and Cdc7-Dbf4 kinases in meiosis [49].

## 2. Separase Structure and Functional Motifs

**Figure 2:**
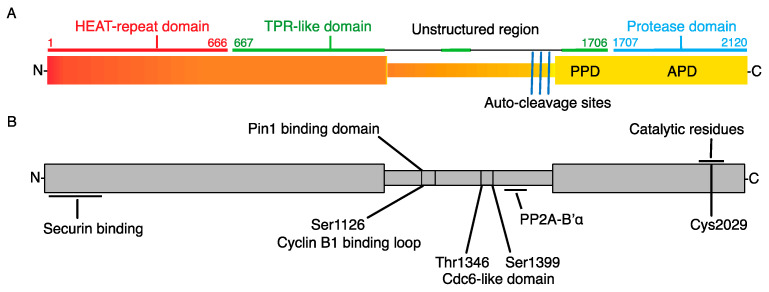
Structural motifs of Separase. (**A**) Human separase consists of super-helical N-terminal and C-terminal domains, which is also called proteolytic domain [50]. The N-terminus consists of a HEAT-repeat domain [50] and a TPR-like domain [49]. The C-terminus contains pseudo protease domain (PPD) and active protease domain (APD) [50,58]. (**B**) The N-terminus contains securin binding site [50] and Pin1 binding domain [56], whose binding depends on Ser1153 residue. The mutation of Ser1126 prevents inhibition of Separase by Cdk1/cyclin B [59]. The unstructured region of separase also contains a cell division cycle 6 (Cdc6)-like domain [60], with two important phosphorylation sites (Thr1346 and Ser1399) [61]. PP2A binds to a 55 amino acids motif (residues 1419–1473). The unstructured domain also contains three Separase auto-cleavage sites (residues Arg1486, Arg1506 and Arg1535 in human Separase) [62,63,64]. The APD contains two conserved residues, a histidine (His2003) and a cysteine (Cys2029) [41]. The function of individual domains is discussed further in the text.

Separase is a large protease from the caspase family, with molecular weight in different organisms ranging from 140 to 240 kDa. The protein (Figure 2) consists of an N-terminal part, which contains ARM and HEAT repeats, and a C-terminal part with an unstructured region in between [50]. The N-terminal part is responsible for the regulation of Separase activity by binding of its inhibitor Securin, and also for the interaction of Separase with substrates. Its length varies between species. The crystal structure of Separase, in complex with its inhibitor Securin, elucidated the mechanism of Securin binding and inhibition [51,52], as well as the details of cohesion cleavage [53], and will be discussed later. Two Separase catalytic domains, of which only the second is active, are located within the C-terminus, which exhibits, in contrast to the N-terminal part, higher conservation between species. The N-terminal and C-terminal parts are separated by an unstructured region, to which another Separase inhibitor, the Cyclin B/CDK1 complex, binds. An unstructured region between the N- and C-terminal parts also serves as a binding site for other proteins (Figure 2) and supports additional Separase functions. Namely, the nuclear export sequence (NES), RG motif and Lys1034 sensitive to sumoylation, which together are crucial for the role of Separase in DNA damage repair [54,55]. The Ser-Pro-containing region of this segment is also important for isomerization by the peptidyl-prolyl isomerase Pin1 [56]. This region also carries residues phosphorylated by Cdk1 [57].

## 3. How Is the Activation of Separase in Mitosis Controlled?

Separation of sister chromatids is an irreversible step. Therefore, several pathways, controlling Separase activity have evolved, ensuring that the enzyme will not activate precociously. It is also important to synchronize the timing of the activation of Separase with other cellular processes, namely with the assembly of the mitotic spindle apparatus and with correct attachment of the chromosomes. The first glimpse of how such a system might work came from experiments with fission [45,65,66] and budding [67,68,69] yeasts. It was shown that the proteins Cut2 or Pds1 are in complex with Separase, and that the destruction of these proteins is required for cohesion cleavage and anaphase entry. Proteins with similar behavior, but no sequence homology to Cut2 or Pds1 from yeasts, were later found in vertebrate species and were called Securins [70]. The mechanism of Separase inhibition by Securin involves one of the Securin domains, which has the ability to serve as a pseudosubstrate of Separase [51,58]. It was further discovered that Securin also carries a conserved LPE motif, similar to the one found in Scc1, which additionally to the pseudosubstrate domain blocks the interaction of Separase with its substrates [71]. The binding of Securin also affects Separase conformation and therefore upon Securin release, Separase undergoes important conformational change, from a trans to cis form, which is imposed by the binding of Peptidyl-prolyl cis/trans isomerase (Pin1). The conformational change is important for the prevention of Securin rebinding and thus for Separase activation [56]. The binding to Separase also has consequences for the stability of Securin. It was shown that the population of Securin in complex with Separase is more stable in comparison to the unbound Securin [72]. The stability of Securin attached to Separase depends on PP2A associated with Separase [62]. Specifically, the PP2A bound to Separase prevents Securin phosphorylation and subsequent destruction and this way increases its stability [73]. The free Securin, on the other hand, is cleared from cells in mitosis by phosphorylation, perhaps by Calcium/calmodulin-dependent protein kinase II (CaMKII), and then by ubiquitination by APC/C [73]. It was shown in mouse oocytes that phenylalanine 125 and 128, which affect Securin stability, are perhaps masked by binding to Separase, and when they are exposed in unbound Securin, they lead into D-box- and KEN-box-dependent Securin destruction [74].

Another well-known inhibitor of Separase is complex of Cyclin B/CDK1 [57]. Additionally, Separase is inhibited by this complex only in vertebrates. Complex of Cyclin B/CDK1 is a master regulator of M phase across species [75,76,77] and its activity was found to be essential for spectacular and dramatic events during mitosis, such as mitotic entry, with disassembly of the nuclear membrane, chromosome condensation and assembly of the spindle apparatus. On the other hand, the inhibition of the activity of this complex is required for chromosome segregation, spindle disassembly and for the exit from mitosis. It was discovered that the activity of Cyclin B/Cdk1 is required for the phosphorylation of serine at position 1126 in human Separase and also for the phosphorylation of several residues within the Cdc6-like domain [57,60]. The phosphorylation of serine 1126, however, represents merely an initial step, required for the binding of Cyclin B/CDK1 to Separase, which then leads into Separase inhibition [59,61]. Serine 1126 phosphorylation is also required for Separase isomerization by Pin1 which, after Securin release, changes the conformation of Separase from a Securin-sensitive into a Securin-resistant form [56]. This model is supported by the observation that more Cyclin B/CDK1 was found in the complex with Separase in cells closer to anaphase [56,72]. The Cyclin B binds to Separase within the Cdc6-like domain [60,61]. In this regard, it is interesting that Cyclin A1, ectopically expressed in oocytes, also induces Separase inhibition, which was abolished by the mutation of S1121A in mouse Separase [78]. This might indicate that the interaction of Separase and Cyclin/CDK is not restricted only to Cyclin B, but other Cyclins might be capable of binding to this site and inhibiting Separase.

Our understanding of the actual mechanism of Separase inhibition by both Securin and Cyclin B/CDK1 was recently significantly improved by cryo-electron microscopy results from Yu and colleagues [58]. They confirmed previous findings that the activity of human Separase is blocked by Securin, acting as pseudosubstrate of Separase [58]. They also discovered that the interaction of the Cyclin B/CDK1/CksI complex with Separase also blocks Separase active site by pseudosubstrate motif, this time, however, provided from the unstructured region of Separase itself. Importantly, the interaction between Separase and Cyclin B/CDK1 also leads into the inactivation of CDK1 by the pseudosubstrate motif also delivered by loop from Separase. The mutual inhibition between Separase and Cyclin B/CDK1 upon binding was discovered earlier [61], however recent work provided important mechanistic details for our understanding of this interaction. It was shown that Separase, in complex with Securin, is sensitive to Cyclin B/CDK1 phosphorylation on serine 1126, but the Cyclin B/CDK1 is unable to bind to Separase, while it is associated with Securin [61].

When Separase is activated, it cleaves not only its targets, such as cohesin, but also itself [63]. The autocleavage plays an important role in the regulation of Separase activity, as well as in the regulation of Cyclin B/CDK binding, and it is specific to vertebrates. Human Separase has three autocleavage sites positioned closely to each other, with sequence motifs similar to the Scc1 cleavage site. It was also shown that even after the cleavage, the fragments remain associated. The biological significance of the autocleavage is not so clear and it is difficult to study this phenomenon since the autocleavage is not required for Separase activity in vitro. However, the effect of abolishing autocleavage has a strong phenotype in in vivo experiments. For example, it was shown that Separase with mutated autocleavage sites delays G2/M transition and chromosome congression [79]. It was also shown that Separase autocleavage impacts its association with PP2A. Additionally, expression of Separase with mutated autocleavage sites caused a loss of sister chromatid cohesion [62]. A recent study addressed further consequences of abolishing Separase autocleavage in vivo [80]. Authors found that the noncleavable Separase is activated much earlier during M phase, but the overall activity is lower in comparison to wild-type Separase. Additionally, abolishing autocleavage reduced Cyclin B/CDK1 binding, in contrast to the cleavage fragments, and cells exhibited increased frequency of chromosome bridges, spindle rocking and overall perturbed chromosome segregation [72].

Recently, a new inhibitory pathway controlling Separase was uncovered [81]. Namely, the inhibition of Separase by binding of Shugoshin 2/Mitotic arrest-deficient protein 2 (Sgo 2/Mad2) complex during the period of activity of the Spindle Assembly Checkpoint (SAC). The quantification of Separase, distributed in complexes with Securin, Sgo2/Mad2 and CDK1, showed 59%, 35% and 6%, respectively. Securin depletion increased the amount of Separase in complex with Sgo 2/Mad2 to 85%. The formation of the inhibitory complex requires prior Mad2 activation, and the complex is abundant in early stages of mitosis, during prometaphase, and it is disassembled in anaphase. The simultaneous deletion of Securin and Sgo2 in interphase cells increased centriole duplication several folds, indicating that the Sgo2/Mad2 complex is also involved in Separase inhibition during interphase. The Sgo2/Mad2 is released from the complex with Separase by Thyroid hormone receptor interactor 13 (TRIP13) and p31comet or by APC/C, which makes even this new axis of Separase inhibition at least partially APC/C-dependent. It will have to be tested whether this pathway is active or sufficient to maintain Separase inactivity in all cell types. In mouse oocytes, for example, it was shown that simultaneous overexpression of Separase with S1121A and T1342A mutations and Securin reduction by morpholino lead into a partial precocious separation of bivalents and sister chromatids in meiosis I [82].

The changes to Separase localization are an important part of the control of its activity. It seems that in interphase, Separase is sequestered from its main substrate, the chromosome cohesion. This is under control of the nuclear export signal sequence motif (NES), in the unstructured region of Separase (Figure 2). In HeLa cells, Separase is excluded from the nucleus during interphase and it is predominantly localized to the cytoplasm [83]. This localization pattern, however, changes upon DNA damage, when the NES is inactivated and the Separase accumulates in the nucleus [55]. In budding yeast, it seems that the transition of the Separase to the nucleus requires Securin [84]. In mitosis, the activity of Separase is restricted to the region in the vicinity of the chromosomes [72].

## 4. How Is Separase Activation Linked to Assembly of the Spindle?

**Figure 3:**
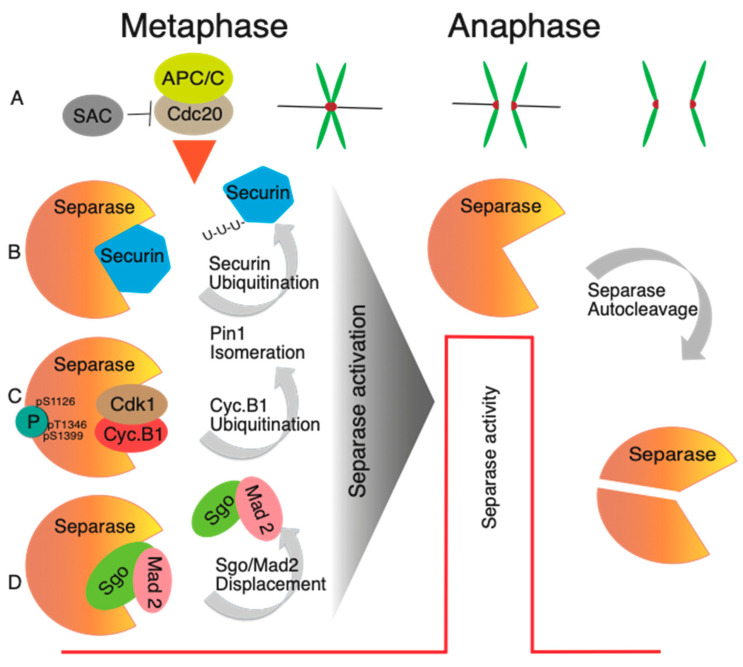
Schematic illustration of mechanisms of Separase inhibition. (**A**) Separase activation is controlled by APC/C activity [57], whose activation is controlled by SAC [92]. After ubiquitination and destruction of Securin and Cyclin B, and disassembly of Sgo2/Mad 2 complex, the Separase is activated and subsequently the sister chromatids separate in anaphase. (**B**,**C**) After activation of APC/C, Securin and Cyclin B ubiquitination is commenced and simultaneously Pin1 isomerase changes the Separase conformation [56]. (**D**) The recently discovered third pathway involves the Sgo2/Mad2 complex, and upon its removal from Separase, after prometaphase and metaphase, the Separase is activated [81]. During the metaphase to anaphase transition, Separase shows abrupt activation and cleaves cohesion. Consequently, the separase undergoes autocleavage [63,64,72].

A period of Separase activity during mitosis requires accurate synchronization with spindle assembly and proper connection and orientation of the chromosomes. The construction of the spindle is monitored by the Spindle Assembly Checkpoint (SAC) pathway (reviewed in [85,86,87]). According to the currently accepted model, during the process of spindle assembly, unattached kinetochores catalyze the conformational change of Mad2 from an open to closed form, which in the cytoplasm then sequesters CDC20 into Mitotic checkpoint complex ((MCC—additional components are Budding uninhibited by benzimidazoles 3 and R1 (Bub3, BubR1), with participation of Aurora B and Monopolar spindle 1 (Mps1) kinases mediating recruitment of Mad1-Mad2 complex to kinetochores), thus preventing activation of APC/C and anaphase entry. Simultaneously, the orientation of sister kinetochores is arranged in a manner that their kinetochores face the opposite spindle poles (amphitelic attachment). This is achieved by tension sensing and correction mechanisms involving Aurora B [88]. Once the proper connection of all kinetochores to the spindle is achieved, the production of MCC ceases and cells are ready for metaphase to anaphase transition. Once the CDC20 is released from MCC, it binds to and activates the APC/C [89,90,91] (reviewed in [92]). This leads into the ubiquitination of multiple substrates, with specific sequence motifs called destruction box [93]. Such motifs are also carried by Separase inhibitors Cyclin B and Securin, and their destruction by APC/C and proteasome leads into full Separase activation and cohesin cleavage. According to the current model of Separase inhibition, Securin and Cyclin B/CDK1 inhibitory pathways are both sensitive to APC/C activity. It seems that the Sgo2/Mad2 pathway is, albeit partially, also sensitive to APC/C [81]. The linkage between the activity of SAC and APC/C and Separase activation ensures that the cohesion cleavage will not be initiated precociously before the correct spindle assembly is achieved (Figure 3); however, there are some exceptions, for example the case of chromosomes with merotelic type of attachment, which evade detection by SAC [94].

## 5. Other Targets Than Cohesin?

**Figure 4:**
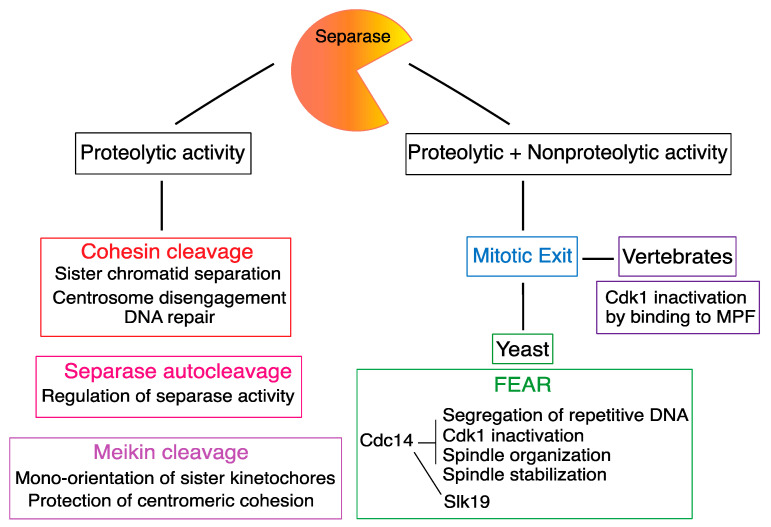
Separase key roles during cell cycle. The proteolytic activity of Separase is required for cohesion and Meikin cleavage and also for autocleavage and centrosome disengagement. For some other roles of Separase, the proteolytic activity is not required, partially for FEAR network in yeasts, although the cleavage of the kinetochore-associated protein Slk19 during anaphase requires Separase proteolytic activity. In vertebrates, Separase is involved in CDK1 inactivation during mitotic exit by formation of a complex with Cyclin B/CDK1 (MPF).

Separase is the only enzyme capable of cohesion cleavage and, therefore, in addition to its role during chromosome segregation, it is also required for cohesion cleavage during DNA repair [55,95,96] (Figure 4). On the other hand, the Scc1 or Rec8 are not the only targets of Separase protease activity and, throughout the time, additional targets carrying similar cleavage motifs were discovered. For example, in budding yeast, Separase was found to be a part of a molecular pathway orchestrating exit from mitosis called FEAR (Cdc Fourteen Early Anaphase Release) network [97,98]. Separase was shown to be important for several tasks within this network, including cleavage of the kinetochore-associated protein Slk19 during anaphase [99], and activation of Cdc14 [97,97]. The FEAR network, however, seems to be specific for budding yeasts, and mammals use different phosphatases to control the exit from mitosis [100]. It was shown that in vertebrates, the Separase participates on exit from mitosis by inactivation of associated Cyclin B/CDK1. This Separase function does not require proteolytic activity and it is based on the formation of a complex with Cyclin B/CDK1 [58,61]. It was proposed that the inhibition of CDK1 activity by Separase is instrumental for poleward movements of chromosomes after cohesion cleavage [72].

It was recently shown that the protein Meiosis-specific kinetochore protein (Meikin) is required for proper chromosome segregation in mouse meiosis I, namely for mono-orientation of sister kinetochores and the protection of centromeric cohesion [101]. Meikin is an essential factor for proper chromosome segregation in meiosis and both males and females are infertile without this protein. Interestingly, Meikin is also a substrate of Separase. Meikin cleavage in meiosis I exposes the centromeric cohesion for cleavage in meiosis II [102]. Meikin carries the same motif ExxR, recognized by Separase in its targets, and mutation of this site prevents Meikin cleavage during meiosis I and causes chromosome alignment defects in meiosis II.

Cell proliferation, proper spindle assembly and chromosome division, are dependent on a correct number of centrosomes per cell [103]. In G1 cells, there is one centrosome inherited from the maternal cell, which consists of two centrioles. During S phase, two new centrioles are formed, eventually giving rise to the new centrosome. This process is very complex and also requires the proteolytic activity of Separase, which was shown to be required for centriole disengagement prior to entry into mitosis [104]. Consistently with participation of Separase, the process of centriole disengagement in late mitosis is blocked by nondegradable Securin. Later, it was shown that Plk1 is also required for centriole disengagement [105]. The precise role of Separase in this process is, however, still not completely understood, namely the cleavage targets. So far, the cohesin ring, as well as pericentrin, were identified as Separase targets during the duplication of centrosomes [106,107]. In Drosophila embryos, however, it seems that the cohesin cleavage alone is not sufficient for centriole disengagement [108].

## 6. Role and Regulation of Separase during Meiosis and Early Development

Early development in mammals is characterized by a high frequency of chromosome segregation errors, which represent the single most frequent case of termination of development in mammals [109]. Separase is a key player in chromosome segregation, and therefore it is conceivable that its regulation might be implicated in the susceptibility of germ cells and embryos to chromosome segregation errors and aneuploidy. In meiosis, Separase is required for the removal of cohesion between sister chromatids, which holds together homologous chromosomes during meiosis I and sister chromatids during meiosis II (reviewed in [110]). It was shown that in mice, the transition from meiosis I to meiosis II is Separase-dependent [38,111,112]. In contrast to the somatic cells, however, the prophase pathway in mammalian female meiosis is suppressed, with the exception of cohesion, which contains Scc1 kleisin subunit, which is also targeted in meiosis [39]. Separase is therefore solely responsible for the removal of cohesion holding bivalents and sister chromatids [38]. Additionally, for accurate segregation of chromosomes in meiosis, the cohesin between sister chromatids has to be preserved until meiosis II, despite the activation of Separase in anaphase I. The protection of centromeric cohesion is accomplished by complex Sgo2/PP2A, which preserves Rec8 against cleavage in the vicinity of the centromeres [113]. How the Separase activity during meiosis is controlled is nevertheless not entirely clear. It was shown that standalone depletion of Securin by morpholino or preventing Cyclin B/CDK1 inhibition by Separase mutation has no effect, it is only when both inhibitory pathways are inhibited simultaneously that the chromosomes separate precociously [101]. This shows that, at least in meiosis I, both inhibitory pathways can fully compensate in case of inhibiting one of them. However, the removal of Securin inhibitor has dire consequences in meiosis II, causing the precocious separation of sister chromatids [114]. The importance of Securin for Separase regulation during meiosis II was supported by the observation that in eggs from aged animals, the Securin levels are lower, which also leads into a precocious separation of sister chromatids before anaphase [115]. Therefore, it seems that in mammalian meiosis, particularly in meiosis II, the regulation of Separase is mostly Securin-based, and although in meiosis I Cyclin B/CDK1 is able to compensate for Securin loss, this is not the case in meiosis II. Moving further in development, it seems that after fertilization, the blastomeres in early embryos become dependent on Separase regulation by Cyclin B/CDK1 [116]. This was tested by replacing endogenous Separase with mutated S1121A, required for Cyclin B/CDK1 binding, which caused failure of early embryonic development. Similarly, it was shown that mouse primordial germ cells also require Separase accessible to Cyclin B/CDK1 binding [116].

## 7. Conclusions and Future Perspectives

From its original discovery as an enzyme which cleaves cohesin, Separase was identified as a key player in multiple events during the cell cycle (Figure 4). Additionally, the most important aspect is certainly the regulation of its enzymatic activity. This can be clearly demonstrated in the case of sister chromatid cohesion. Precocious separation of sister chromatids, before the spindle apparatus is fully matured, would unavoidably lead into uneven distribution of genetic information between daughter cells and finally into cell loss, or tumorigenesis, in multicellular organisms [117]. Therefore, in higher eukaryotes, multiple pathways exist, which inhibit the Separase activity until the right time. Importantly, all three known inhibitory pathways are, at least partially, sensitive to the APC/C activity. This ensures that the Separase will be activated only after SAC is satisfied, and not precociously before accurate spindle assembly. At the same time, removal of Separase inhibition before anaphase by the powerful activity of APC/C is essential for preventing chromosome nondisjunction, which is another problem that cells might face during anaphase, and which will also cause aneuploidy. The redundancy in pathways inhibiting Separase in vertebrate cells is essential, since it provides necessary plasticity for cells to achieve correct chromosome segregation in various conditions. Although the Separase inhibitory pathways are abundant, experiments using transgenic animals showed that there might be specific cell types which are more dependent on one certain pathway for its inhibition. For example, in case of whole-body knockout of Securin, the fetal fibroblasts showed defects in cell proliferation and division [118,119]. This demonstrates that for this cell type, Securin might be indispensable; in the case of regulation by Cyclin B/CDK1, the female germ cells and early embryos seems to be affected when Separase is resistant to this pathway [116].

Despite indisputable recent progress in our understanding of Separase inhibition, there are still open questions requiring more experimental work. Some of these efforts should be aimed at studying the importance of each type of regulation in specific cells and tissues within the organism, while others can address the differences between results obtained by live cell assays and biochemical studies [80]. Additionally, it will be also important to resolve how the different pathways inhibiting Separase are coordinated temporally and spatially as cells are progressing throughout mitosis. It is also equally important to address the significance of inhibition of CDK1 by Separase during the exit from mitosis. Although it seems that in prometaphase and metaphase only a small portion of Separase is engaged in such a complex [81], its importance increases towards anaphase, when the levels of Securin and Cyclin B become reduced by APC/C-proteasome machinery and a rapid inhibition of the remaining CDK1 activity is required [72].

Separase is an essential enzyme, and it might be important as a potential target for anticancer therapy, and additionally its expression might also serve as a cancer prognosis marker [120,121,122,123,124,125]. In the past few years, several promising inhibitors of Separase were identified, based on in vitro compound screening using Rad21 cleavage assay [120,124,125]. The independent screens revealed several small molecules, called Separase inhibitor or Sepin 1 or Separase Inhibitory Compounds (SIC) 1, 3, 5 and 6 (Table 1), which were subsequently characterized further. The design of such inhibitors is by no means trivial. The first problem is that the potential Separase pharmaceutical inhibitors should discriminate between separase and other caspases. Then, there is a problem with the residual activity of separase, which might be sufficient for the execution of sister chromatid separation in tissue culture cells [120] or might contribute to the resistance of tumors with higher separase levels in vivo [124]. Despite the potential problems surrounding the first generation of Separase inhibitors, this area of research is very important. Future work should be directed towards procedures making cells susceptible to Separase inhibition in case of lower expression, as well as characterizing molecular mechanisms responsible for the inhibition of cellular proliferation [125].

## Figures and Tables

**Table 1:** Separase inhibitors—Separase is overexpressed in many cancer cells and its deregulated activity might lead into aneuploidy. Since the activity of Separase is essential for cell division, it also represents a potential chemotherapeutic target for anticancer drugs, such as Sepin-1 or Separase Inhibitory Compounds (SIC) 1, 3, 5 and 6 [120,123,124].

Inhibitor	Effect	References
Sepin-1	Identified by Rad21 cleavage in vitro, good in vitro inhibitory effect and selective in vivo inhibition, molecular mechanisms of inhibition involve transcription factor FoxM1.	[124,125]
SIC1, 3, 5	Identified by Rad21 cleavage in vitro, the in vivo activity requires lower Separase levels.	[120]
SIC5−6	Improved in vitro inhibitory effect.	[120]

## Data Availability

Not applicable.

## Abbreviations

APC/C	Anaphase-promoting complex/cyclosome
APD	Active protease domain
ARM	Armadillo repeat
BUB	Budding uninhibited by benzimidazoles
CaMKII	Calcium/calmodulin-dependent protein kinase II
Cdc14	Cell division cycle 14
CDC20	Cell division cycle 20
Cdc7-dbf4	Cell division cycle 7-related protein kinase, DBF4-dependent kinase
Cdh1	Cdc20 homolog 1
CDK	Cyclin-dependent kinase
CksI	Cyclin-dependent protein kinase regulatory subunit I
CSF	Cytostatic factor
D-box	Destruction box
DNA	Deoxyribonucleic acid
ESPl1	Extra spindle poles-like protein 1
FEAR	Fourteen early anaphase release
MAD	Mitotic arrest-deficient protein
MAU2	MAU2 sister chromatid cohesion factor
MCC	Mitotic checkpoint complex
MEIKIN	Meiosis-specific kinetochore protein
MPS1	Monopolar spindle 1 kinase
NES	Nuclear export sequence
NIPBL	Nipped-B-like protein
Pds	Precocious dissociation of sisters
Pin1	Peptidyl-prolyl cis/trans isomerase
Plk1	Polo-like kinase 1
PP2A	Protein phosphatase 2A
PPD	Pseudo protease domain
Rec 8	Meiotic recombination protein Rec8
RNA	Ribonucleic acid
SAC	Spindle assembly checkpoint
SCC	Sister chromatid cohesion protein
SGO	Shugoshin
SMC	Structural maintenance of chromosomes
Slk19	Kinetochore-associated protein Slk19
TRIP13	Thyroid hormone receptor interactor 13
Wapl	Wings apart-like protein

## References

[1] Michaelis C., Ciosk R., Nasmyth K. (1997). Cohesins: Chromosomal proteins that prevent premature separation of sister chromatids. Cell.

[2] Guacci V., Koshland D., Strunnikov A. (1997). A direct link between sister chromatid cohesion and chromosome condensation revealed through the analysis of MCD1 in S. cerevisiae. Cell.

[3] Haering C.H., Farcas A.M., Arumugam P., Metson J., Nasmyth K. (2008). The cohesin ring concatenates sister DNA molecules. Nature.

[4] Nasmyth K., Haering C.H. (2009). Cohesin: Its roles and mechanisms. Annu. Rev. Genet..

[5] Peters J.M., Nishiyama T. (2012). Sister chromatid cohesion. Cold Spring Harb. Perspect. Biol..

[6] Morales C., Losada A. (2018). Establishing and dissolving cohesion during the vertebrate cell cycle. Curr. Opin. Cell Biol..

[7] Yatskevich S., Rhodes J., Nasmyth K. (2019). Organization of Chromosomal DNA by SMC Complexes. Annu. Rev. Genet..

[8] Perea-Resa C., Wattendorf L., Marzouk S., Blower M.D. (2021). Cohesin: Behind Dynamic Genome Topology and Gene Expression Reprogramming. Trends Cell Biol..

[9] Davidson I.F., Peters J.M. (2021). Genome folding through loop extrusion by SMC complexes. Nat. Rev. Mol. Cell Biol..

[10] Higashi T.L., Uhlmann F. (2022). SMC complexes: Lifting the lid on loop extrusion. Curr. Opin. Cell. Biol..

[11] Hartman T., Stead K., Koshland D., Guacci V. (2000). Pds5p is an essential chromosomal protein required for both sister chromatid cohesion and condensation in Saccharomyces cerevisiae. J. Cell Biol..

[12] Losada A., Yokochi T., Kobayashi R., Hirano T. (2000). Identification and Characterization of Sa/Scc3p Subunits in the Xenopus and Human Cohesin Complexes. J. Cell Biol..

[13] Kueng S., Hegemann B., Peters B.H., Lipp J.J., Schleiffer A., Mechtler K., Peters J.M. (2006). Wapl controls the dynamic association of cohesin with chromatin. Cell.

[14] Haering C.H., Nasmyth K. (2003). Building and breaking bridges between sister chromatids. Bioessays.

[15] Makrantoni V., Marston A.L. (2018). Cohesin and chromosome segregation. Curr. Biol..

[16] Ciosk R., Shirayama M., Shevchenko A., Tanaka T., Toth A., Shevchenko A., Nasmyth K. (2000). Cohesin’s binding to chromosomes depends on a separate complex consisting of Scc2 and Scc4 proteins. Mol. Cell.

[17] Gillespie P.J., Hirano T. (2004). Scc2 couples replication licensing to sister chromatid cohesion in Xenopus egg extracts. Curr. Biol..

[18] Tonkin E.T., Wang T.J., Lisgo S., Bamshad M.J., Strachan T. (2004). NIPBL, encoding a homolog of fungal Scc2-type sister chromatid cohesion proteins and fly Nipped-B, is mutated in Cornelia de Lange syndrome. Nat. Genet..

[19] Watrin E., Schleiffer A., Tanaka K., Eisenhaber F., Nasmyth K., Peters J.M. (2006). Human Scc4 is required for cohesin binding to chromatin, sister-chromatid cohesion, and mitotic progression. Curr. Biol..

[20] Rankin S., Ayad N.G., Kirschner M.W. (2005). Sororin, a substrate of the anaphase-promoting complex, is required for sister chromatid cohesion in vertebrates. Mol. Cell.

[21] Ladurner R., Kreidl E., Ivanov M.P., Ekker H., Idarraga-Amado M.H., Busslinger G.A., Wutz G., Cisneros D.A., Peters J.M. (2016). Sororin actively maintains sister chromatid cohesion. EMBO J..

[22] Sundin O., Varshavsky A. (1981). Arrest of segregation leads to accumulation of highly intertwined catenated dimers: Dissection of the final stages of SV40 DNA replication. Cell.

[23] Murray A.W., Schultes N.P., Szostak J.W. (1986). Chromosome length controls mitotic chromosome segregation in yeast. Cell.

[24] Farcas A.M., Uluocak P., Helmhart W., Nasmyth K. (2011). Cohesin’s concatenation of sister DNAs maintains their intertwining. Mol. Cell.

[25] Coelho P.A., Queiroz-Machado J., Sunkel C.E. (2003). Condensin-dependent localisation of topoisomerase II to an axial chromosomal structure is required for sister chromatid resolution during mitosis. J. Cell Sci..

[26] Charbin A., Bouchoux C., Uhlmann F. (2014). Condensin aids sister chromatid decatenation by topoisomerase II. Nucleic. Acids Res..

[27] Piskadlo E., Oliveira R.A. (2017). A Topology-Centric View on Mitotic Chromosome Architecture. Int. J. Mol. Sci..

[28] Chu L., Zhang Z., Mukhina M., Zickler D., Kleckner N. (2022). Sister chromatids separate during anaphase in a three-stage program as directed by interaxis bridges. Proc. Natl. Acad. Sci. USA.

[29] Waizenegger I.C., Hauf S., Meinke A., Peters J.M. (2000). Two distinct pathways remove mammalian cohesin from chromosome arms in prophase and from centromeres in anaphase. Cell.

[30] Haarhuis J.H., Elbatsh A.M., Rowland B.D. (2014). Cohesin and its regulation: On the logic of X-shaped chromosomes. Dev. Cell.

[31] Tedeschi A., Wutz G., Huet S., Jaritz M., Wuensche A., Schirghuber E., Davidson I.F., Tang W., Cisneros D.A., Bhaskara V. (2013). Wapl is an essential regulator of chromatin structure and chromosome segregation. Nature.

[32] Kitajima T.S., Kawashima S.A., Watanabe Y. (2004). The conserved kinetochore protein shugoshin protects centromeric cohesion during meiosis. Nature.

[33] Rabitsch K.P., Gregan J., Schleiffer A., Javerzat J.P., Eisenhaber F., Nasmyth K. (2004). Two fission yeast homologs of Drosophila Mei-S332 are required for chromosome segregation during meiosis I and II. Curr. Biol..

[34] Salic A., Waters J.C., Mitchison T.J. (2004). Vertebrate shugoshin links sister centromere cohesion and kinetochore microtubule stability in mitosis. Cell.

[35] Kitajima T.S., Sakuno T., Ishiguro K., Iemura S., Natsume T., Kawashima S.A., Watanabe Y. (2006). Shugoshin collaborates with protein phosphatase 2A to protect cohesin. Nature.

[36] Riedel C.G., Katis V.L., Katou Y., Mori S., Itoh T., Helmhart W., Gálová M., Petronczki M., Gregan J., Cetin B. (2006). Protein phosphatase 2A protects centromeric sister chromatid cohesion during meiosis I. Nature.

[37] Marston A.L. (2015). Shugoshins: Tension-sensitive pericentromeric adaptors safeguarding chromosome segregation. Mol. Cell Biol..

[38] Kudo N.R., Wassmann K., Anger M., Schuh M., Wirth K.G., Xu H., Helmhart W., Kudo H., McKay M., Maro B. (2006). Resolution of chiasmata in oocytes requires separase-mediated proteolysis. Cell.

[39] Silva M.C.C., Powell S., Ladstätter S., Gassler J., Stocsits R., Tedeschi A., Peters J.M., Tachibana K. (2020). Wapl releases Scc1-cohesin and regulates chromosome structure and segregation in mouse oocytes. J. Cell Biol..

[40] Uhlmann F., Lottspeich F., Nasmyth K. (1999). Sister-chromatid separation at anaphase onset is promoted by cleavage of the cohesin subunit Scc1. Nature.

[41] Uhlmann F., Wernic D., Poupart M.A., Koonin E.V., Nasmyth K. (2000). Cleavage of cohesin by the CD clan protease separin triggers anaphase in yeast. Cell.

[42] Hauf S., Waizenegger I.C., Peters J.M. (2001). Cohesin cleavage by separase required for anaphase and cytokinesis in human cells. Science.

[43] Wirth K.G., Wutz G., Kudo N.R., Desdouets C., Zetterberg A., Taghybeeglu S., Seznec J., Ducos G.M., Ricci R., Firnberg N. (2006). Separase: A universal trigger for sister chromatid disjunction but not chromosome cycle progression. J. Cell Biol..

[44] Baum P., Yip C., Goetsch L., Byers B. (1988). A yeast gene essential for regulation of spindle pole duplication. Mol. Cell Biol..

[45] Uzawa S., Samejima I., Hirano T., Tanaka K., Yanagida M. (1990). The fission yeast cut1+ gene regulates spindle pole body duplication and has homology to the budding yeast ESP1 gene. Cell.

[46] Buonomo S.B., Clyne R.K., Fuchs J., Loidl J., Uhlmann F., Nasmyth K. (2000). Disjunction of homologous chromosomes in meiosis I depends on proteolytic cleavage of the meiotic cohesin Rec8 by separin. Cell.

[47] Alexandru G., Uhlmann F., Mechtler K., Poupart M.A., Nasmyth K. (2001). Phosphorylation of the cohesin subunit Scc1 by Polo/Cdc5 kinase regulates sister chromatid separation in yeast. Cell.

[48] Hauf S., Roitinger E., Koch B., Dittrich C.M., Mechtler K., Peters J.M. (2005). Dissociation of cohesin from chromosome arms and loss of arm cohesion during early mitosis depends on phosphorylation of SA2. PLoS Biol..

[49] Katis V.L., Lipp J.J., Imre R., Bogdanova A., Okaz E., Habermann B., Mechtler K., Nasmyth K., Zachariae W. (2010). Rec8 phosphorylation by casein kinase 1 and Cdc7-Dbf4 kinase regulates cohesin cleavage by separase during meiosis. Dev. Cell.

[50] Viadiu H., Stemmann O., Kirschner M.W., Walz T. (2005). Domain structure of separase and its binding to securin as determined by EM. Nat. Struct. Mol. Biol..

[51] Luo S., Tong L. (2017). Molecular mechanism for the regulation of yeast separase by securin. Nature.

[52] Boland A., Martin T.G., Zhang Z., Yang J., Bai X.C., Chang L., Scheres S.H., Barford D. (2017). Cryo-EM structure of a metazoan separase-securin complex at near-atomic resolution. Nat. Struct. Mol. Biol..

[53] Lin Z., Luo X., Yu H. (2016). Structural basis of cohesin cleavage by separase. Nature.

[54] Sun Y., Kucej M., Fan H.Y., Yu H., Sun Q.Y., Zou H. (2009). Separase is recruited to mitotic chromosomes to dissolve sister chromatid cohesion in a DNA-dependent manner. Cell.

[55] Hellmuth S., Gutiérrez-Caballero C., Llano E., Pendás A.M., Stemmann O. (2018). Local activation of mammalian separase in interphase promotes double-strand break repair and prevents oncogenic transformation. EMBO J..

[56] Hellmuth S., Rata S., Brown A., Heidmann S., Novak B., Stemmann O. (2015). Human chromosome segregation involves multi-layered regulation of separase by the peptidyl-prolyl-isomerase Pin1. Mol. Cell.

[57] Stemmann O., Zou H., Gerber S.A., Gygi S.P., Kirschner M.W. (2001). Dual inhibition of sister chromatid separation at metaphase. Cell.

[58] Yu J., Raia P., Ghent C.M., Raisch T., Sadian Y., Cavadini S., Sabale P.M., Barford D., Raunser S., Morgan D.O. (2021). Structural basis of human separase regulation by securin and CDK1-cyclin B1. Nature.

[59] Holland A.J., Taylor S.S. (2006). Cyclin-B1-mediated inhibition of excess separase is required for timely chromosome disjunction. J. Cell Sci..

[60] Boos D., Kuffer C., Lenobel R., Körner R., Stemmann O. (2008). Phosphorylation-dependent binding of cyclin B1 to a Cdc6-like domain of human separase. J. Biol. Chem..

[61] Gorr I.H., Boos D., Stemmann O. (2005). Mutual inhibition of separase and Cdk1 by two-step complex formation. Mol. Cell.

[62] Holland A.J., Böttger F., Stemmann O., Taylor S.S. (2007). Protein phosphatase 2A and separase form a complex regulated by separase autocleavage. J. Biol. Chem..

[63] Waizenegger I., Giménez-Abián J.F., Wernic D., Peters J.M. (2002). Regulation of human separase by securin binding and autocleavage. Curr. Biol..

[64] Zou H., Stemman O., Anderson J.S., Mann M., Kirschner M.W. (2002). Anaphase specific auto-cleavage of separase. FEBS Lett..

[65] Funabiki H., Kumada K., Yanagida M. (1996). Fission yeast Cut1 and Cut2 are essential for sister chromatid separation, concentrate along the metaphase spindle and form large complexes. EMBO J..

[66] Funabiki H., Yamano H., Kumada K., Nagao K., Hunt T., Yanagida M. (1996). Cut2 proteolysis required for sister-chromatid seperation in fission yeast. Nature.

[67] Yamamoto A., Guacci V., Koshland D. (1996). Pds1p is required for faithful execution of anaphase in the yeast, Saccharomyces cerevisiae. J. Cell Biol..

[68] Cohen-Fix O., Peters J.M., Kirschner M.W., Koshland D. (1996). Anaphase initiation in Saccharomyces cerevisiae is controlled by the APC-dependent degradation of the anaphase inhibitor Pds1p. Genes Dev..

[69] Ciosk R., Zachariae W., Michaelis C., Shevchenko A., Mann M., Nasmyth K. (1998). An ESP1/PDS1 Complex Regulates Loss of Sister Chromatid Cohesion at the Metaphase to Anaphase Transition in Yeast. Cell.

[70] Zou H., McGarry T.J., Bernal T., Kirschner M.W. (1999). Identification of a vertebrate sister-chromatid separation inhibitor involved in transformation and tumorigenesis. Science.

[71] Rosen L.E., Klebba J.E., Asfaha J.B., Ghent C.M., Campbell M.G., Cheng Y., Morgan D.O. (2019). Cohesin cleavage by separase is enhanced by a substrate motif distinct from the cleavage site. Nat. Commun..

[72] Shindo N., Kumada K., Hirota T. (2012). Separase sensor reveals dual roles for separase coordinating cohesin cleavage and cdk1 inhibition. Dev. Cell.

[73] Hellmuth S., Böttger F., Pan C., Mann M., Stemmann O. (2014). PP2A delays APC/C-dependent degradation of separase-associated but not free securin. EMBO J..

[74] Thomas C., Wetherall B., Levasseur M.D., Harris R.J., Kerridge S.T., Higgins J.M.G., Davies O.R., Madgwick S. (2021). A prometaphase mechanism of securin destruction is essential for meiotic progression in mouse oocytes. Nat. Commun..

[75] Kishimoto T. (2018). MPF-based meiotic cell cycle control: Half a century of lessons from starfish oocytes. Proc. Jpn. Acad. Ser. B.

[76] Crncec A., Hochegger H. (2019). Triggering mitosis. FEBS Lett..

[77] Holder J., Poser E., Barr F.A. (2019). Getting out of mitosis: Spatial and temporal control of mitotic exit and cytokinesis by PP1 and PP2A. FEBS Lett..

[78] Radonova L., Pauerova T., Jansova D., Danadova J., Skultety M., Kubelka M., Anger M. (2020). Cyclin A1 in Oocytes Prevents Chromosome Segregation and Anaphase Entry. Sci. Rep..

[79] Papi M., Berdougo E., Randall C.L., Ganguly S., Jallepalli P.V. (2005). Multiple roles for separase auto-cleavage during the G2/M transition. Nat. Cell Biol..

[80] Shindo N., Kumada K., Iemura K., Yasuda J., Fujimori H., Mochizuki M., Tamai K., Tanaka K., Hirota T. (2022). Autocleavage of separase suppresses its premature activation by promoting binding to cyclin B1. Cell Rep..

[81] Hellmuth S., Gómez-H L., Pendás A.M., Stemmann O. (2020). Securin-independent regulation of separase by checkpoint-induced shugoshin-MAD2. Nature.

[82] Chiang T., Schultz R.M., Lampson M.A. (2011). Age-dependent susceptibility of chromosome cohesion to premature separase activation in mouse oocytes. Biol. Reprod..

[83] Sun Y., Yu H., Zou H. (2006). Nuclear exclusion of separase prevents cohesin cleavage in interphase cells. Cell Cycle.

[84] Hornig N.C., Knowles P.P., McDonald N.Q., Uhlmann F. (2002). The dual mechanism of separase regulation by securin. Curr. Biol..

[85] Musacchio A. (2015). The Molecular Biology of Spindle Assembly Checkpoint Signaling Dynamics. Curr. Biol..

[86] Lara-Gonzalez P., Pines J., Desai A. (2021). Spindle assembly checkpoint activation and silencing at kinetochores. Semin. Cell Dev. Biol..

[87] Foley E.A., Kapoor T.M. (2013). Microtubule attachment and spindle assembly checkpoint signalling at the kinetochore. Nat. Rev. Mol. Cell Biol..

[88] McVey S.L., Cosby J.K., Nannas N.J. (2021). Aurora B Tension Sensing Mechanisms in the Kinetochore Ensure Accurate Chromosome Segregation. Int. J. Mol. Sci..

[89] Sudakin V., Ganoth D., Dahan A., Heller H., Hershko J., Luca F.C., Ruderman J.V., Hershko A. (1995). The cyclosome, a large complex containing cyclin-selective ubiquitin ligase activity, targets cyclins for destruction at the end of mitosis. Mol. Biol. Cell.

[90] Hartwell L.H., Culotti J., Reid B. (1970). Genetic control of the cell-division cycle in yeast. I. Detection of mutants. Proc. Natl. Acad. Sci. USA.

[91] King R.W., Peters J.M., Tugendreich S., Rolfe M., Hieter P., Kirschner M.W. (1995). A 20S complex containing CDC27 and CDC16 catalyzes the mitosis-specific conjugation of ubiquitin to cyclin B. Cell.

[92] Peters J.M. (2006). The anaphase promoting complex/cyclosome: A machine designed to destroy. Nat. Rev. Mol. Cell Biol..

[93] King R.W., Glotzer M., Kirschner M.W. (1996). Mutagenic analysis of the destruction signal of mitotic cyclins and structural characterization of ubiquitinated intermediates. Mol. Biol. Cell.

[94] Gregan J., Polakova S., Zhang L., Tolić-Nørrelykke I.M., Cimini D. (2011). Merotelic kinetochore attachment: Causes and effects. Trends Cell Biol..

[95] Nagao K., Adachi Y., Yanagida M. (2004). Separase-mediated cleavage of cohesin at interphase is required for DNA repair. Nature.

[96] McAleenan A., Clemente-Blanco A., Cordon-Preciado V., Sen N., Esteras M., Jarmuz A., Aragón L. (2013). Post-replicative repair involves separase-dependent removal of the kleisin subunit of cohesin. Nature.

[97] Stegmeier F., Visintin R., Amon A. (2002). Separase, polo kinase, the kinetochore protein Slk19, and Spo12 function in a network that controls Cdc14 localization during early anaphase. Cell.

[98] D’Amours D., Amon A. (2004). At the interface between signaling and executing anaphase--Cdc14 and the FEAR network. Genes Dev..

[99] Sullivan M., Lehane C., Uhlmann F. (2001). Orchestrating anaphase and mitotic exit: Separase cleavage and localization of Slk19. Nat. Cell Biol..

[100] Wurzenberger C., Gerlich D.W. (2011). Phosphatases: Providing safe passage through mitotic exit. Nat. Rev. Mol. Cell Biol..

[101] Kim J., Ishiguro K., Nambu A., Akiyoshi B., Yokobayashi S., Kagami A., Ishiguro T., Pendas A.M., Takeda N., Sakakibara Y. (2015). Meikin is a conserved regulator of meiosis-I-specific kinetochore function. Nature.

[102] Maier N.K., Ma J., Lampson M.A., Cheeseman I.M. (2021). Separase cleaves the kinetochore protein Meikin at the meiosis I/II transition. Dev. Cell.

[103] Nigg E.A. (2007). Centrosome duplication: Of rules and licenses. Trends Cell Biol..

[104] Tsou M.F., Stearns T. (2006). Mechanism limiting centrosome duplication to once per cell cycle. Nature.

[105] Tsou M.F., Wang W.J., George K.A., Uryu K., Stearns T., Jallepalli P.V. (2009). Polo kinase and separase regulate the mitotic licensing of centriole duplication in human cells. Dev. Cell.

[106] Schöckel L., Möckel M., Mayer B., Boos D., Stemmann O. (2011). Cleavage of cohesin rings coordinates the separation of centrioles and chromatids. Nat. Cell Biol..

[107] Matsuo K., Ohsumi K., Iwabuchi M., Kawamata T., Ono Y., Takahashi M. (2012). Kendrin is a novel substrate for separase involved in the licensing of centriole duplication. Curr. Biol..

[108] Oliveira R.A., Nasmyth K. (2013). Cohesin cleavage is insufficient for centriole disengagement in Drosophila. Curr. Biol..

[109] Hassold T., Hunt P. (2001). To err (meiotically) is human: The genesis of human aneuploidy. Nat. Rev. Genet..

[110] Petronczki M., Siomos M.F., Nasmyth K. (2003). Un ménage à quatre: The molecular biology of chromosome segregation in meiosis. Cell.

[111] Terret M.E., Wassmann K., Waizenegger I., Maro B., Peters J.-M., Verlhac M.-H. (2003). The Meiosis I-to-Meiosis II Transition in Mouse Oocytes Requires Separase Activity. Curr. Biol..

[112] Herbert M., Levasseur M., Homer H., Yallop K., Murdoch A., McDougall A. (2003). Homologue disjunction in mouse oocytes requires proteolysis of securin and cyclin B1. Nat. Cell Biol..

[113] Wassmann K. (2022). Separase Control and Cohesin Cleavage in Oocytes: Should I Stay or Should I Go. Cells.

[114] Nabti I., Reis A., Levasseur M., Stemmann O., Jones K.T. (2008). Securin and not CDK1/cyclin B1 regulates sister chromatid disjunction during meiosis II in mouse eggs. Dev. Biol..

[115] Nabti I., Grimes R., Sarna H., Marangos P., Carroll J. (2017). Maternal age-dependent APC/C-mediated decrease in securin causes premature sister chromatid separation in meiosis II. Nat. Commun..

[116] Huang X., Andreu-Vieyra C.V., Wang M., Cooney A.J., Matzuk M.M., Zhang P. (2009). Preimplantation mouse embryos depend on inhibitory phosphorylation of separase to prevent chromosome missegregation. Mol. Cell Biol..

[117] Levine M.S., Holland A.J. (2018). The impact of mitotic errors on cell proliferation and tumorigenesis. Genes Dev..

[118] Mei J., Huang X., Zhang P. (2001). Securin is not required for cellular viability, but is required for normal growth of mouse embryonic fibroblasts. Curr. Biol..

[119] Wang Z., Yu R., Melmed S. (2001). Mice lacking pituitary tumor transforming gene show testicular and splenic hypoplasia, thymic hyperplasia, thrombocytopenia, aberrant cell cycle progression, and premature centromere division. Mol. Endocrinol..

[120] Henschke L., Frese M., Hellmuth S., Marx A., Stemmann O., Mayer T.U. (2019). Identification of Bioactive Small Molecule Inhibitors of Separase. ACS Chem. Biol..

[121] Spiess B., Kleiner H., Flach J., Fabarius A., Saussele S., Hofmann W.K., Seifarth W. (2020). Separase activity distribution can be a marker of major molecular response and proliferation of CD34+ cells in TKI-treated chronic myeloid leukemia patients. Ann. Hematol..

[122] Jo M., Kusano Y., Hirota T. (2021). Unraveling pathologies underlying chromosomal instability in cancers. Cancer Sci..

[123] Gurvits N., Löyttyniemi E., Nykänen M., Kuopio T., Kronqvist P., Talvinen K. (2017). Separase is a marker for prognosis and mitotic activity in breast cancer. Br. J. Cancer.

[124] Zhang N., Scorsone K., Ge G., Kaffes C.C., Dobrolecki L.E., Mukherjee M., Lewis M.T., Berg S., Stephan C.C., Pati D. (2014). Identification and Characterization of Separase Inhibitors (Sepins) for Cancer Therapy. J. Biomol. Screen.

[125] Zhang N., Pati D. (2018). Separase Inhibitor Sepin-1 Inhibits Foxm1 Expression and Breast Cancer Cell Growth. J. Cancer Sci. Ther..

